# Mometasone and desloratadine additive effect on eosinophil survival and cytokine secretion from epithelial cells

**DOI:** 10.1186/1465-9921-12-23

**Published:** 2011-02-27

**Authors:** Joaquim Mullol, Francisco de Borja Callejas, Maria Asunción Martínez-Antón, Eva Méndez-Arancibia, Isam Alobid, Laura Pujols, Antonio Valero, Cesar Picado, Jordi Roca-Ferrer

**Affiliations:** 1 IDIBAPS, Hospital Clínic. CIBER de Enfermedades Respiratorias (CIBERES), Villarroel 170, Barcelona, 08036, Catalonia, Spain; 2 Rhinology Unit & Smell Clinic, ENT, Hospital Clínic. CIBER de Enfermedades Respiratorias (CIBERES), Villarroel 170, Barcelona, 08036, Catalonia, Spain; 3 Pneumology Departments, Hospital Clínic. CIBER de Enfermedades Respiratorias (CIBERES), Villarroel 170, Barcelona, 08036, Catalonia, Spain

## Abstract

**Background:**

Although antihistamines and topical corticosteroids are used in combination to treat allergic rhinitis, their additive effect has not been yet demonstrated. The aim was investigate the antiinflammatory additive effect of mometasone and desloratadine on cytokine and sICAM-1 secretion by epithelial cells, and on eosinophil survival stimulated by human epithelial cells secretions from nasal mucosa and polyps.

**Methods:**

Epithelial cells obtained from nasal mucosa or polyps were stimulated with 10% fetal bovine serum in presence of mometasone (10^-11^M-10^-5^M) with/without desloratadine (10^-5^M). Cytokine and sICAM-1 concentrations in supernatants were measured by ELISA. Peripheral blood eosinophils were incubated during 4 days with epithelial cell secretions with (10^-11^M-10^-5^M) and/or desloratadine (10^-5^M) and survival assessed by Trypan blue. Results are expressed as percentage (mean ± SEM) compared to control.

**Results:**

Fetal bovine serum stimulated IL-6, IL-8, GM-CSF and sICAM-1 secretion. In mucosa and polyp epithelial cells, mometasone inhibited this induced secretion while desloratadine inhibited IL-6 and IL-8. The combination of 10^-5^M desloratadine and 10^-9^M mometasone reduced IL-6 secretion (48 ± 11%, p < 0.05) greater extent than mometasone alone (68 ± 10%) compared to control (100%). Epithelial cell secretions induced eosinophil survival from day 1 to 4, this effect being inhibited by mometasone. At day 4, the combination of mometasone (10^-11^M) and desloratadine (10^-5^M) provoked an increased inhibition of eosinophil survival induced by cell secretions (27 ± 5%, p < 0.01) than mometasone (44 ± 7%) or desloratadine (46 ± 7%) alone.

**Conclusions:**

These results suggest that the combination of desloratadine and mometasone furoate have a greater antinflammatory effect in an in vitro model of eosinophil inflammation than those drugs administered alone.

## Background

Allergic rhinitis (AR) and chronic rhinosinusitis (CRS) with/without nasal polyposis (NP) are diseases characterized by upper airway mucosal inflammation with elevated levels of pro-inflammatory cytokines and eosinophil infiltration [1-3]. Concentrations of IL-1β, IL-4, and IL-5 are increased in nasal secretions from patients with AR, while other cytokines such as IL-6, IL-8, eotaxin, tumor necrosis factor-alpha (TNF-α), Interferon-γ, granulocyte-macrophage colony-stimulating factor (GM-CSF), vascular endothelial growth factor (VEGF) and transforming growth factor-β (TGF-β), as well as chemokines such as eotaxin and RANTES, are also increased in patients suffering from CRS with NP [4-6].

The increased level of pro-inflammatory mediators plays a role in the eosinophil infiltration of nasal mucosa. Some of these cytokines and other mediators such as platelet-activating factor (PAF), adhesion molecules and cysteinyl leukotrienes induce eosinophilopoeisis, cell recruitment from peripheral blood to the site of inflammation, and increase eosinophil survival and activation [7-12]. In fact, we have previously demonstrated that upper-airway epithelial cells may contribute to eosinophilic inflammation through the release of GM-CSF, IL-8 and TNF-α [7].

The first line treatment of upper airway inflammation includes corticosteroids and antihistamines [2]. Corticosteroids such as mometasone furoate (MF) and others have been shown to be effective in the treatment of allergic rhinitis and rhinosinusitis [13,14]. Among others, the antiinflammatory effect of costicosteroids includes the inhibition of cytokine secretion from epithelial cells and the reduction of the eosinophil survival [7,11,15,16]. Specifically, it has been reported that MF inhibits the synthesis of several cytokines in both the respiratory cell line A549 [17] and human keratinocytes [18], but the effect on upper airway epithelial cells has not been yet reported. In addition, it has been demonstrated that MF induces eosinophil apoptosis [19] and reduces their number in NM biopsies [20], suggesting that, like other glucocorticoids, MF is capable to directly act on these cells. On the other hand, antihistamines such as desloratadine (DL) have shown to be effective in the treatment of allergic rhinitis and asthma [21,22], including some anti-inflammatory functions. In fact, DL inhibits cytokine secretion from NM and NP epithelial cells, basophils, and mast cells [12,23,24].

Although current ARIA Guidelines recommend the combination of antihistamines and topical corticosteroids for the treatment of allergic rhinitis [25], few studies have been conducted to demonstrate the efficacy of combined treatment on upper airway inflammation, and the benefits compared with antihistamine or corticosteroid monotherapy are still not clear. In fact, some studies with patients suffering from AR demonstrated no additional benefit when MF where used in combination with loratadine [26], while others found significant improvement when using the combination of flunisolide with loratadine [27], fluticasone with cetirizine [28] and MF with DL [29]. However, no study has yet reported the additive anti-inflammatory effect of antihistamines and corticosteriods.

The present study, carried out in on in vitro validated model of cultured upper-airway epithelial cells and peripheral blood eosinophils [7,11,12,15,16], was designed to investigate the additive anti-inflammatory effects of DL and MF on proinflammatory cytokines and soluble intercellular adhesion molecule (sICAM)-1 secretion from both NM and NP epithelial cell cultures as well as on eosinophil survival primed by secretions from both NM and NP cultured epithelial cells.

## Methods

### Materials

Ham's F-12 and RPMI 1640 medium was purchased from Bio Whittaker Europe (Verviers, Belgium); 24-well culture plates from Costar (Cultek SL, Madrid, Spain); desloratadine and mometasone furoate from Schering Plough (New Jersey, USA); penicillin-streptomycin, fetal bovine serum (FBS) from Invitrogen Corporation (Paisley, Scotland, UK); and amphotericin B from Squibb (Esplugues de Llobregat, Catalonia, Spain). Hydrocortisone, N-formyl-methionyl-leucyl-phenylalanine, human transferrin, bovine insulin, 3,3',5-triiodo-l-tyrosine sodium salt, protease type XIV, light mineral oil, glutamine, trypan blue and dimethyl sulfoxide (DMSO) were obtained from Sigma-Aldrich Co. (Madrid, Spain); endothelial cell growth supplement and epidermal growth factor were supplied by Collaborative Research Inc. (Bedfort, MA, USA); cytokine ELISA kits from Amersham Biosciences Europe (Cerdanyola, Spain) and Diaclone (Stamford, CT, USA); and rat tail collagen type I from Upstate (Lake Placid, NY, USA).

### Study population

Nasal mucosa specimens were obtained from 9 patients (7 men, 2 women), ranging in age from 23 to 62 years (45.5 ± 5.2 yr), who underwent nasal corrective surgery for septal dismorphy, turbinate hypertrophy, or both. Skin-prick test was positive in two patients (22.2%). None of the patients were receiving topical or systemic glucocorticoid or antihistamine treatment on the 4 week prior the surgery.

Nasal polyp specimens were obtained from 9 patients (5 men, 4 women), ranging from 34 to 83 years (56.9 ± 5.2 yr), underwent endoscopic sinonasal surgery with nasal polypectomy. Skin-prick test was positive in two patients (22.2%). Three patients (33.3%) also had concomitant asthma and 6 patients (66.6%) were on regular treatment with intranasal corticosteroids. None of them had aspirin sensitivity.

None of the patients had had an upper airway infection the 2 weeks before surgery.

All patients gave informed consent to participate in the study, which was approved by the Scientific and Ethic's Committee of our Institution.

### Isolation of epithelial cells

Tissue specimens were placed in Ham's F-12 medium supplemented with penicillin (100 UI/ml), streptomycin (100 μg/ml), amphotericin B (2 μg/ml), and immediately transported to the laboratory. Epithelial cells from nasal mucosa or polyps were isolated by protease digestion using a technique previously reported [7,8,15,16]. Viability of cells was assessed by trypan blue dye exclusion using a hemocytometer. Cell population was characterized using smears obtained by cytocentrifugation (500 rpm, 10 min) and stained with May-Grünwald-Giemsa or with mouse monoclonal anti-cytoketarin antibody using the immune-alkaline phosphatase method [7,8,15]. After tissue protease digestion, cell viability was 91.8 ± 2.9% for NMs and 89.0 ± 2.9% for NPs, and the percentage of epithelial cell purity was 98.9 ± 0.1% for NM and 92.3 ± 1.3% for NP specimens.

### Culture of epithelial cells

Epithelial cell suspensions (10^5 ^cells/well) were placed on 24-well plates coated with rat tail collagen type I in a hormonally defined serum-free media: F-12 culture medium (2 ml), antibiotics (penicillin, 100 UI/ml; streptomycin, 100 μg/ml), amphotericin B (2 μg/ml), glutamine (150 μg/ml), transferrin (5 μg/ml), insulin (5 μg/ml), epidermal growth factor (25 ng/ml), endothelial cell growth factor supplement (15 μg/ml), triiodothyronine (200 pM) and hydrocortisone (100 nM). Epithelial cells were cultured in 5% CO2 humidified atmosphere at 37°C, the culture media being changed every 2 days. After cell culture, the percentage of epithelial cell purity was 100% for both NM and NP cultures.

Generation of Human Epithelial Conditioned Media (HECM). When epithelial cell cultures reached 80% confluence, the hormonally defined serum-free media was switched to RPMI-1640 media supplemented with antibiotics (penicillin, 100 UI/ml; streptomycin, 100 μg/ml), amphotericin B (2 μg/ml), glutamine (150 μg/ml) and HEPES buffer (25 nM). Since previous studies have shown that non-stimulated epithelial cells produce low levels of cytokines [7,15], cultured epithelial cells were incubated with FBS at 10% in the presence or absence of different concentrations of MF (from 10^-11 ^to 10^-5^M) and/or DL (10^-5^M) for 24 hours. After incubation, cell supernatants (HECM) were harvested from cultures, centrifuged at 400 g (10 min, 25°C), sterilized through 0.22 μm filters, and stored at -80°C until used. In order to avoid different effects of FBS from different batches and sources, the same manufacturing batch was used in all experiments.

### Enzymo-Linked Immunoassays (ELISA) of cytokines and sICAM-1

The concentrations of GM-CSF, IL-6, IL-8, and sICAM-1 were measured in HECM from NM and NP cultured epithelial cells using commercial ELISA kits. The assay ranges were: 15.4 to 600 pg/ml for GM-CSF, 1.56 to 50 pg/ml for IL-6, 25 to 1000 pg/ml for IL-8, and 250 to 8000 pg/ml for sICAM-1. To verify that the substances used in the different experiments (MF, DL, FBS) did not affect the ELISA results, wells containing either culture media alone or media with the highest drug concentration used in the different protocols were compared (n = 3). None of the substances showed any intrinsic effect on the ELISA final values.

### Isolation of eosinophils

Normodense eosinophils were obtained from 10 atopic and non-atopic subjects with >3% in peripheral blood eosinophils. None of the patients were receiving topical or systemic glucocorticoid or antihistamine treatment on the 4 week prior the blood extraction. All patients gave informed consent to participate in the study, which was approved by the Scientific and Ethic's Committee of our Institution. Eosinophils were obtained by a previously described method [8,9,12,16] using discontinuous Percoll^® ^gradients. Eosinophil viability and purity (> 90%) were quantified by trypan blue dye exclusion and May-Grünwald-Giemsa staining, respectively.

### Assessment of Eosinophil survival

Eosinophils (2.5 × 10^5 ^cells/well) were incubated in 24-well tissue culture plates with RPMI (2 ml) in the presence or absence of MF (from 10^-11 ^to 10^-5^M) and/or DL (10^-5^M) at 37°C for 1 hr before the addition of 10% HECM from nasal mucosa (NM-HECM) or polyps (NP-HECM). Eosinophil survival index was assessed at 24 hr (day 1), 48 hr (day 2), 72 hr (day 3) and 96 hr (day 4) of incubation by trypan blue dye exclusion. The eosinophil survival index was calculated as follows: number of eosinophils recovered × percentage of eosinophil viability/number of eosinophils delivered on day 0. In order to reduce the variability of HECM in all experiments, nasal mucosa or nasal polyp HECM were created by mixing cell supernatants from all NM or NP epithelial cell cultures.

Because MF and DL were diluted in ethanol and DMSO, respectively, and the HECM added to the eosinophil cultures contained 10% FBS, we investigated the effect of ethanol, DMSO and FBS on eosinophil survival. Neither ethanol, DMSO nor FBS at the higher final concentration present in the culture media (0.1% ethanol when MF was at 10^-5^M, 0.1% DMSO when DL was at 10^-5^M, and 1% FBS when HECM was at 10%) had a significant effect on eosinophil survival (data not shown).

### Statistical Analysis

Statistical evaluations were performed using the statistical software Microsoft SPSS 16.0. Results are expressed as mean ± SEM (standard error of the mean). A non-parametric test, Wilcoxon's signed-rank test was used in cytokine secretion experiments and U Mann-Whitney test was used for statistical comparisons in eosinophil survival experiments. P < 0.05 was considered statistically significant.

## Results

### Effect of FBS on cytokine and sICAM-1 secretion

In NM epithelial cell cultures (N = 9), FBS increased the secretion of IL-6 (media: 254 ± 65 pg/ml; 10% FBS: 1697 ± 437 pg/ml; p < 0.05), IL-8 (media: 1504 ± 462 pg/ml; 10% FBS: 5186 ± 1132 pg/ml; p < 0.05), GM-CSF (media: 159 ± 56 pg/ml; 10% FBS: 395 ± 115 pg/ml; p < 0.05) and sICAM-1 (media: 517 pg/ml ± 147 pg/ml; 10% FBS: 1606 ± 320 pg/ml; p < 0.05).

In NP epithelial cell cultures (N = 9), FBS increased the secretion of IL-6 (media: 376 ± 207 pg/ml; 10% FBS: 2132 ± 779 pg/ml; p < 0.05), IL-8 (media: 1252 ± 836 pg/ml; 10% FBS: 4420 ± 2852 pg/ml; p < 0.05), GM-CSF (media: 115 ± 23 pg/ml; 10% FBS: 393 ± 118 pg/ml; p < 0.05) and sICAM-1 (media: 498 ± 108 pg/ml; 10% FBS: 2111 ± 751 pg/ml; p < 0.05).

No significant differences were found in the concentration of these cytokines and sICAM-1 between NM and NP-HECM.

### Effect of mometasone furoate on cytokine and sICAM-1 secretion

**Figure 1:**
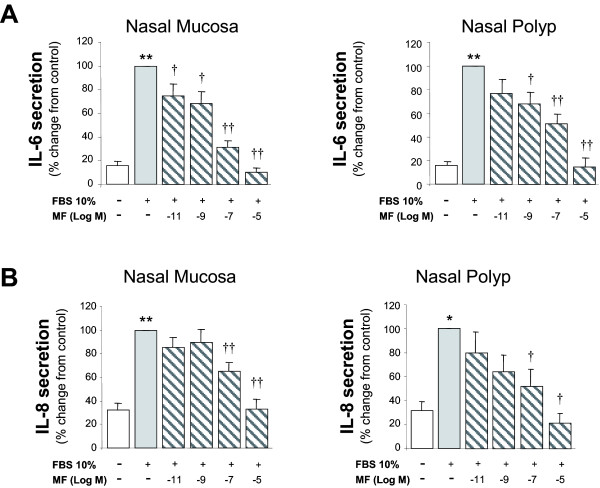
**Effect of mometasone on IL-6 and IL-8 induced secretion from epithelial cells**. Epithelial cells were incubated with 10% fetal bovine serum (FBS) in presence of mometasone (MF) for 24 h. MF caused a dose-related inhibitory effect on FBS-induced IL-6 (A) and IL-8 (B) release. Results expressed as mean ± SEM. Wilcoxon Signed Rank test was used for statistical comparison. * P < 0.05 and ** P < 0.01 compared to control; † P < 0.05 and †† P < 0.01 compared to 10% FBS. N = 9.

**Figure 2:**
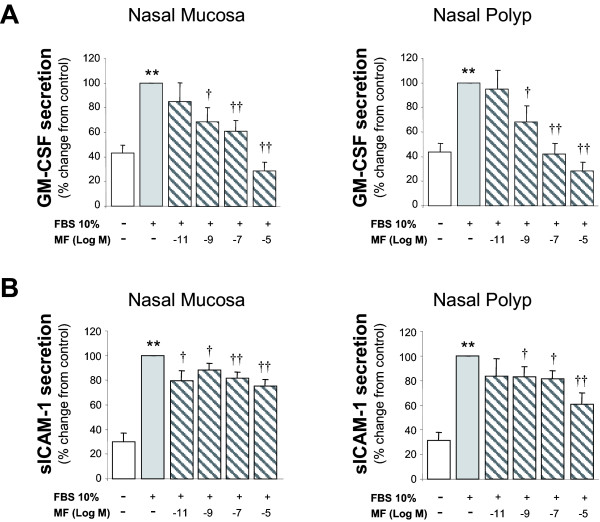
**Effect of mometasone on GM-CSF and sICAM-1 induced secretion from epithelial cells**. Epithelial cells were incubated with 10% fetal bovine serum (FBS) in presence of mometasone (MF) for 24 h. MF caused a dose-related inhibitory effect on FBS-induced GM-CSF (A) sICAM-1 (B) release. Results expressed as mean ± SEM. Wilcoxon Signed Rank test was used for statistical comparison. ** P < 0.01 compared to control; †P < 0.05 and †† P < 0.01 compared to 10% FBS. N = 9.

In both NM and NP polyp epithelial cell cultures, MF significantly decreased the FBS-induced IL-6, IL-8 and GM-CSF secretion in a dose-dependent manner. MF also inhibited the sICAM-1 secretion in NM and NP epithelial cell cultures, but not in a dose-response manner. Compared to NM, MF effect was less potent in NP cultures for the secretion of IL-6 and sICAM-1 (Figures 1 and 2).

### Combined effect of mometasone furoate plus desloratadine on cytokine and sICAM-1 secretion

**Figure 3:**
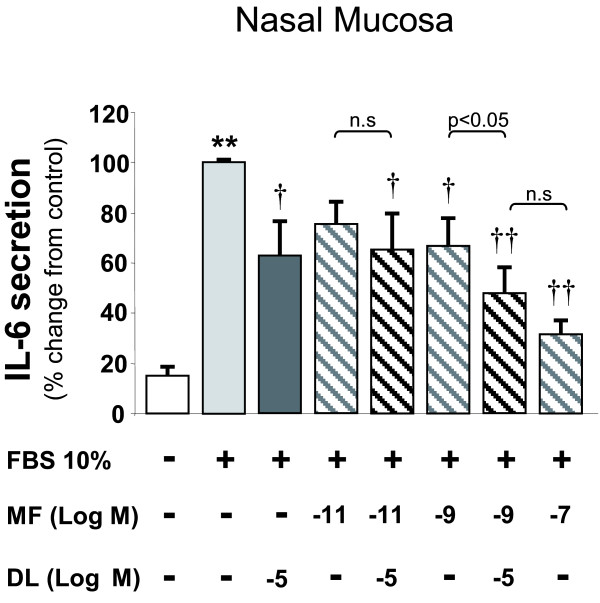
**Additive effect of mometasone and desloratadine on interleukin-6 secretion from nasal mucosa epithelial cells**. Nasal mucosa epithelial cell were incubated with 10% fetal bovine serum (FBS) in presence of mometasone (MF) with/without desloratadine (DL). Additive effect was found when using MF at 10^-9^M plus DL 10^-5^M. Results expressed as mean ± SEM. Wilcoxon Signed Rank test was used for statistical comparison. ** P < 0.01 compared to control; † P < 0.05 and †† P < 0.01 compared to 10% FBS. N = 9.

In NM cultured epithelial cells (N = 9), and compared to FBS treated cultures (100%), the combination of DL at 10^-5^M and MF at 10^-9^M reduced the FBS-induced IL-6 release (48 ± 11%) significantly higher than when MF at 10^-9^M (68 ± 10%) was used alone (Figure 3) and not significantly different from MF at 10^-7^M (32 ± 6%) or DL at 10^-5^M (62 ± 13%). The inhibitory effect of DL (10^-5^M) plus MF (10^-11 ^to 10^-7^M) on IL-6 release in NP epithelial cells were not significantly different when compared to that of MF alone (data not shown).

In NP cultured epithelial cells (N = 9), and compared to FBS treated cultures (100%), the combination of DL at 10^-5^M and MF at 10^-11^M decreased significantly the FBS-induced sICAM-1 secretion (68 ± 10%, p < 0.05), whereas both drugs administered alone did not. The inhibitory effect of DL (10^-5^M) plus MF (10^-11 ^to 10^-7^M) on IL-6 release in NM epithelial cells were not significantly different when compared to that of MF alone (data not shown).

The inhibitory effect of DL (10^-5^M) plus MF (10^-11 ^to 10^-7^M) on IL-8 and GM-CSF release in both NM and NP epithelial cells were not significantly different when compared to that of MF alone (data not shown).

### Effect of mometasone furoate on HECM-induced eosinophil survival

**Figure 4:**
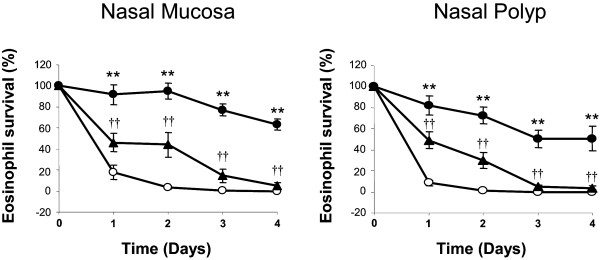
**Kinetics of human epithelial conditioned media (HECM) and mometasone effect on eosinophil survival**. HECM at 10% (filled circles) from nasal mucosa and nasal polyps significantly increased eosinophil survival compared to control media (open circles) from day 1 to 4. Mometasone at 10^-5 ^M (filled triangles) inhibited the HECM-induced eosinophil survival at all time points. Results expressed as mean ± SEM of eosinophil survival index. Mann-Whitney U test was used for statistical comparison ** p < 0.01, HECM compared to control media; †† P < 0.01 MF compared to HECM. N = 10.

**Figure 5:**
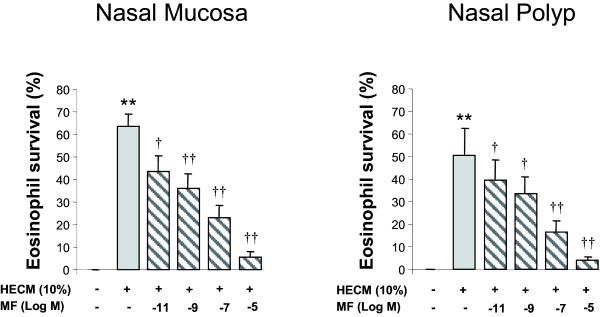
**Mometasone effect on eosinophil survival at day 4**. Human epithelial conditioned media (HECM) at 10% (dark grey bars) from nasal mucosa and nasal polyp induced eosinophil survival. Mometasone (MF, light grey bars) caused a dose-related inhibition of the induced eosinophil survival. Results expressed as mean ± SEM of eosinophil survival index. Wilcoxon Signed Rank test was used for statistical comparison. ** p < 0.01, compared to control media; † p < 0.05, †† p < 0.01, compared to HECM.

HECM from both NM and NP significantly (p < 0.01) induced eosinophil survival from day 1 to day 4 compared to media alone (N = 10). MF at 10^-5^M significantly decreased the NM and NP-HECM-induced eosinophil survival from day 1 to day 4 (Figure 4). At day 4, MF had a significant dose-related inhibitory effect (from 10^-5 ^to 10^-11^M) on both the NM and NP-HECM-induced eosinophil survival (Figure 5).

### Additive effect of mometasone furoate plus desloratadine on eosinophil survival

**Figure 6:**
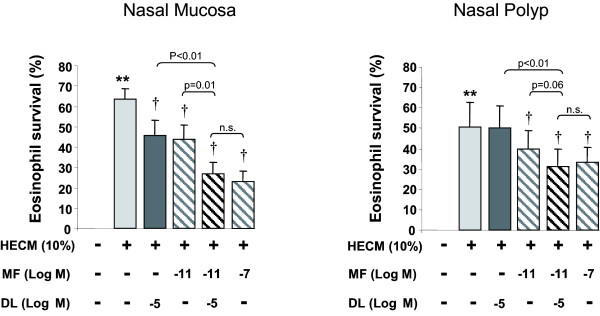
**Additive effect of mometasone and desloratadine on eosinophil survival at day 4**. Human epithelial conditioned media (HECM) at 10% (dark grey bars) from nasal mucosa and nasal polyp increased eosinophil survival compared. The combination of mometasone (MF) at 10^-11^M and desloratadine (DL) at 10^-5^M (striped bars) caused an inhibitory effect higher than MF (light grey bars) or DL (white bars) alone, and similar to MF at 10^-7^M. This additive effect was significant when eosinophil survival was induced by nasal mucosa HECM. Results expressed as mean ± SEM of eosinophil survival index. Wilcoxon Signed Rank test was used for statistical comparison. ** p < 0.01, compared to control media; † p < 0.05, compared to HECM. N = 10.

There were no differences between MF and the combination with DL when studied NM and NP-HECM-induced eosinophil survival at days 1 to 3. At day 4, the combination of MF at 10^-11^M and DL at 10^-5^M caused a significant decrease in NM-HECM-induced eosinophil survival that was higher that when MF or DL where used alone, and similar to the effect of MF at 10^-7^M alone. When eosinophil survival was induced by NP-HECM, the combination of MF at 10^-11^M and DL at 10^-5^M also caused a significant decrease in HECM-induced eosinophil survival that was significantly higher than DL alone but not for MF alone, although at tendency was found (p = 0.06). This effect was also similar to that from MF 10^-7^M (Figure 6).

## Discussion

The main findings of our study are: 1st) fetal bovine serum induced the secretion of IL-6, IL-8, GM-CSF, and sICAM-1 by cultured epithelial cells from both nasal mucosa and polyps; 2nd) in both nasal mucosa and polyp epithelial cells, mometasone inhibited the induced secretion of IL-6, IL-8, GM-CSF and sICAM-1; 3rd) desloratadine weakly but significantly potentiated the inhibitory effect of low concentrations of mometasone (nanomolar) on IL-6 secretion from nasal mucosa epithelial cells; 4th) epithelial cell secretions from both nasal mucosa and nasal polyps induced eosinophil survival; 5th) mometasone inhibited in a dose-dependent manner the eosinophil survival induced by both nasal mucosa and nasal polyp epithelial secretions; and 6th) desloratadine weakly but significantly potentiated the effect of low concentrations of mometasone (picomolar) on decreasing eosinophil survival, especially when epithelial secretions were from nasal mucosa.

In the present study, we have shown that human nasal mucosa and polyp epithelial cells increased the release of IL-6, IL-8, GM-CSF, and sICAM-1 in response to FBS. These findings confirm our previous studies which show that cultured nasal epithelial cells from both human nasal mucosa and nasal polyp express and release GM-CSF, IL-1β, IL-6, IL-8 and TNF-α [7,11,12,15,16,30]. Moreover, a recent study has reported high concentrations of IL-6 in nasal tissue from patients suffering from CRS with NP [6]

In our in vitro model of eosinohil inflammation, MF showed an inhibitory effect on FBS-induced IL-6, IL-8, GM-CSF, and sICAM-1 secretion in both nasal mucosa and polyp epithelial cell cultures. In fact, we have previously demonstrated that other corticosteroids, such as beclometasone dipropionate fluticasone, triamcinolone and budesonide, have a similar effect [11,15,16]. This inhibitory effect suggests that these drugs, including MF may decrease inflammation in the upper airways by inhibiting pro-inflammatory cytokine release by epithelial cells, and consequently, leading to a reduction in inflammatory cell recruitment and activation promoted by such cytokines. In support with our findings, it has been reported that MF inhibit the cytokine-induced GM-CSF expression in a respiratory cell line [17], the LPS-induced IL-1, IL-6 and TNF-α expression in murine blood cells [18] as well as the ICAM-1 expression in skin [31] and lung fibroblasts [32].

In the present study, DL inhibited IL-6 secretion from both NM and NP epithelial cells. We realize that the high concentrations of DL used in our study are significantly higher than those found in blood or epithelial lining fluid during the treatment of patients. However, since our research is a mechanistic study, our research cannot be used as a guide for therapeutic indications. In fact, previous studies have found similar results in relation to DL effect on pro-inflammatory mediators' production and secretion, not only in epithelial cells but also in other cell types. On this regard, DL decreased IL-6 and IL-8 secretion from basophilic cells (KU812) and human mast cell line (HMC-1) [33], IL-4 and IL-13 from basophil-enriched suspension [24], and GM-CSF secretion from HMC-1 cells [34] and airway epithelial cells [12]. The inhibition of a wide range of cytokines suggests that DL may play a role in modulating mediators associated with the airway inflammatory process.

When used in combination with the corticosteroid MF, DL was able to increase the inhibitory effect caused by MF alone on IL-6 secretion, and to inhibit IL-8 secretion in a dose of MF that caused no significant effect on this cytokine when administered alone. Thus, DL seems to improve and potentiate MF effects on cytokine secretion by nasal epithelial cells. However, this effect seems to be present only at low but significant inhibitory doses of MF. To some extent, these findings agree with clinical trials in which it has been demonstrated improvements in the sneezing [27], rhinorrhoea [27], total symptom score [28], nasal itching [28] and total nasal symptom score [29] when combining different corticosteroids and antihistamines in the treatment of allergic and non-allergic rhinitis.

In the present study, MF decreased the eosinophil survival induced by epithelial secretions from both NM and NP. In keeping with our results, it has been reported that MF reduced the sputum eosinophilia in asthmatic patients [35], decreased the number of eosinophils in nasal mucosa biopsies [20] and induced apoptosis in eosinophil cultured in vitro [19]. In addition, we found that DL reduced eosinophil survival induced by epithelial secretions from nasal mucosa, as previously reported [12].

When investigating the combined effect of MF plus DL on eosinophil viability, an additive effect was found on eosinophil survival induced by epithelial cell secretions, since DL increased the inhibitory effect of MF alone. In the same line, it has been reported that loratadine improved the effect of a corticosteroid in the treatment of non-allergic rhinitis with eosinophilia decreasing eosinophil counts in nasal smears [27] and nasal sneezing in seasonal allergic rhinitis [26].

## Conclusions

In summary, the present study suggests that the combination of mometasone furoate and desloratadine diminish eosinophil inflammation in a greater extent than those drugs administered alone, confirming a common antiinflammatory mechanism for these kind of drugs. However, further in vivo study must be performed to clarify the clinical applications of the in vitro findings.

## Abbreviations

AR: allergic rhinitis; CRS: chronic rhinosinusitis; DL: desloratadine; DMSO: dimethyl sulfoxide; FBS: fetal bovine serum; GM-CSF: granulocyte-macrophage colony-stimulating factor; HECM: human epithelial conditioned media; MF: mometasone fuorate; NP: nasal polyposis; PAF: platelet-activating factor; sICAM-1: soluble intercellular adhesion molecule-1; TGF-β; transforming growth factor-β; TNF-α: tumor necrosis factor-α; VEGF: vascular endothelial growth factor.

## Competing interests

JM: In the last 5 years has acted as member of National and International Scientific Advisory Boards for UCB Pharchim, Uriach SA, Schering Plough, GSK, MSD, and Zambon; has been awarded with grants for Research Projects from Schering-Plough, Uriach SA, UCB Pharchim, and MSD; and participated as investigator in Clinical Trials for UCB Farma, FAES, Uriach SA, Schering-Plough, and GSK.

AV, in the last 5 years has acted as member of National International Scientific Advisory Boards for UCB, Uriach SA, Schering Plough, GSK, MSD; has been awarded with grants for Research Projects from Schering-Plough, Uriach SA, UCB, and MSD; and participated as investigator in Clinical Trials for FAES, Uriach SA.

CP has been awarded with grants for Research Projects from Uriach SA, Phadia, Chiesi, AstraZeneca, Leti, and MSD; and participated as investigator in Clinical Trials for Uriach SA and Chiesi.

The rest of the authors declare that they have no competing interests.

## Authors' contributions

JM, MAMA, CP and JRF conceived the study, planned the overall experimental design and wrote the manuscript; MAMA, EMA, LP and FBC perform the cell Cultures, ELISA measurements and assessment of eosinophil survival; EMA, LP, FBC and AV participated to the conception of the project, interpretation of data; IA carried out the patient selection and obtaining of surgical specimens; AV participated in the patient selection. All authors read and approved the final manuscript.
